# Delayed Recognition of Deterioration of Patients in General Wards Is Mostly Caused by Human Related Monitoring Failures: A Root Cause Analysis of Unplanned ICU Admissions

**DOI:** 10.1371/journal.pone.0161393

**Published:** 2016-08-18

**Authors:** Louise S. van Galen, Patricia W. Struik, Babiche E. J. M. Driesen, Hanneke Merten, Jeroen Ludikhuize, Johannes I. van der Spoel, Mark H. H. Kramer, Prabath W. B. Nanayakkara

**Affiliations:** 1 Department of Internal Medicine, Section Acute Medicine, VU University Medical Center, Amsterdam, The Netherlands; 2 Department of Public and Occupational Health, EMGO Institute for Health and Care Research, VU University Medical Centre, Amsterdam, The Netherlands; 3 Department of Anaesthesiology, Academic Medical Center, Amsterdam, the Netherlands; 4 Department of Intensive Care, VU University Medical Center, Amsterdam, The Netherlands; Azienda Ospedaliero Universitaria Careggi, ITALY

## Abstract

**Background:**

An unplanned ICU admission of an inpatient is a serious adverse event (SAE). So far, no in depth-study has been performed to systematically analyse the root causes of unplanned ICU-admissions. The primary aim of this study was to identify the healthcare worker-, organisational-, technical,- disease- and patient- related causes that contribute to acute unplanned ICU admissions from general wards using a Root-Cause Analysis Tool called PRISMA-medical. Although a Track and Trigger System (MEWS) was introduced in our hospital a few years ago, it was implemented without a clear protocol. Therefore, the secondary aim was to assess the adherence to a Track and Trigger system to identify deterioration on general hospital wards in patients eventually transferred to the ICU.

**Methods:**

Retrospective observational study in 49 consecutive adult patients acutely admitted to the Intensive Care Unit from a general nursing ward. 1. PRISMA-analysis on root causes of unplanned ICU admissions 2. Assessment of protocol adherence to the early warning score system.

**Results:**

Out of 49 cases, 156 root causes were identified. The most frequent root causes were healthcare worker related (46%), which were mainly failures in monitoring the patient. They were followed by disease-related (45%), patient-related causes (7, 5%), and organisational root causes (3%). In only 40% of the patients vital parameters were monitored as was instructed by the doctor. 477 vital parameter sets were found in the 48 hours before ICU admission, in only 1% a correct MEWS was explicitly documented in the record.

**Conclusions:**

This in-depth analysis demonstrates that almost half of the unplanned ICU admissions from the general ward had healthcare worker related root causes, mostly due to monitoring failures in clinically deteriorating patients. In order to reduce unplanned ICU admissions, improving the monitoring of patients is therefore warranted.

## Introduction

Despite steady improvement in hospital care and programs to reduce harm in hospitalised patients, serious adverse events (SAEs) are still common [1–4]. In one out of ten patients, the occurrence of an SAE during their admission contributes to permanent disability or to death [5]. Previous studies have documented that some adverse events are probably preventable and that they are often related to errors in management [4]. An unplanned admission to the Intensive Care Unit (ICU) of an inpatient from a general ward is considered as a SAE. According to the NICE (National Intensive Care Evaluation) criteria, an unplanned ICU admission is defined as an admission that could not have been deferred without risk for at least 12 hours [6]. It is known that unexpected ICU admission leads to a poor long term survival, especially in older patients [7].

Hence, it is important to detect deteriorating patients timely and treat them early in order to prevent an eventual ICU admission or to transfer them to the ICU on time to improve clinical outcomes. One of the ways to achieve this is by implementing a Rapid Response System (RRS) [8, 9]. These systems, composed of an afferent and efferent limb, are specifically designed to enable early recognition and management of deteriorating patients on general wards. The efferent limb consists of trained ICU personnel forming a Rapid Intervention Team (RIT) who deliver immediate treatment to deteriorating patients at the bedside after being called in by ward clinical staff. The clinical staff can detect patients deterioration early by using a Track and Trigger Systems (TTS) such as Modified Early Warning Score (MEWS) [10, 11]. This afferent limb plays a key-role in limiting unplanned ICU admission, since early detection of deteriorating patients could potentially prevent this.

However, the effectiveness of these TTSs in preventing unplanned ICU admissions remains unclear [8, 12]. In addition, (root) causes leading to delayed detection of these deteriorating patients in the wards are mostly unknown. The circumstances leading to delayed detection of deteriorating patients in the wards are probably multi-causal. Current literature has identified certain patient characteristics associated with unplanned admissions into the ICU, such as age, and having a surgical procedure [13]. Also, certain iatrogenic events such as disease induced by a drug prescribed or environmental events (falls) have shown to cause ICU admissions [14, 15]. These studies, however, tell comparatively little about the healthcare worker related and organisational/system related root causes, which, if known, can be used to improve early detection of deteriorating patients and potentially avoid unplanned ICU-admissions.

A useful tool analysing these types of root causes is the PRISMA-tool (Prevention and Recovery Information System for Monitoring and Analysis). The main goal of the PRISMA method is to build a database of incidents and process deviations by creating causal trees, from which conclusions may be drawn to suggest optimal countermeasures. This in-depth analysis method has been accepted by the World Alliance for Patient Safety of the World Health Organisation and has shown to provide effective starting points for improvement in quality of care [16–19].

No study has yet been performed to systematically analyse and identify the root causes leading to late detection and treatment of deteriorating patients in wards. To formulate possible improvement and prevention strategies with the aim of reducing the number of ICU admissions insight into these causes is essential. Also, an optimally implemented RRS could contribute to increased patient safety by recognising those in need of extra care without any unnecessary delay.

Therefore, the main aims of this retrospective record review study were to (1.) analyse the healthcare worker-, organisational-, technical-, disease- and patient- related causes that contribute to late detection and treatment of deteriorating patients in the general wards using PRISMA-medical analysis, and (2.) assess the adherence to and effectiveness of an already implemented TTS on general wards in the early recognition of deteriorating patients transferred to the ICU.

## Materials and Methods

### Study design

This retrospective, record review study included unplanned ICU admissions from general wards in the VU University Medical Center in Amsterdam (VUmc), The Netherlands. This is an academic 733-bedded medical center with approximately 50,000 admissions per year. The Adult Intensive Care Unit consists of 22 beds.

We decided to include the first 50 consecutive patient records of 2015 meeting the inclusion criteria to explore the causes of unplanned ICU-admissions, since previous studies have shown that around 50 PRISMA-analyses are credible and provide a well-founded causal-profile [16, 19]. The following criteria were used for inclusion: all patients on general wards aged 18 years and older who were admitted to the ICU unplanned according to the NICE criteria (“an admission that could not have been deferred without risk for at least 12 hours”) [6]. Only the first ICU admission of the patient was included. Excluded from the study sample were: patients admitted on the ICU immediately from the emergency department, the operation room, medium care, high care, or coronary care; patients transferred from other hospitals; patients with a planned ICU admission (i.e. after surgery). The Ethics committee of VU University Medical Center, Amsterdam, approved the study and necessity for informed consent was waived. Information from the clinical records was anonymized and de-identified prior to analysis.

**Fig 1:**
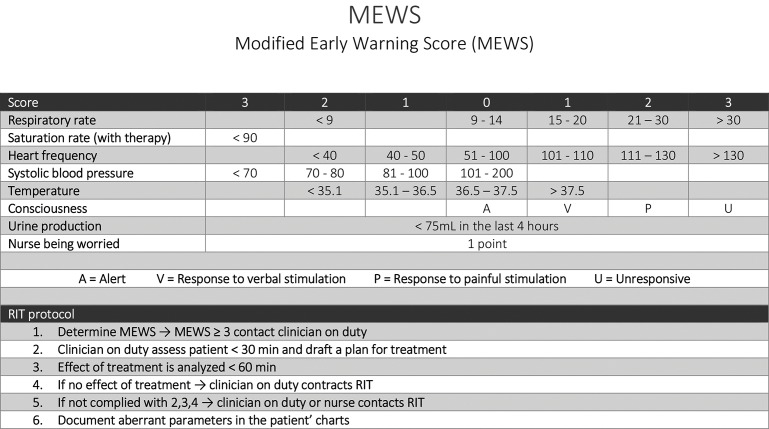
MEWS protocol in VUmc.

The RRS used in the VUmc is the MEWS, validated by Subbe et al. in 2007, which was implemented in the VUmc in 2014 [11]. The MEWS used at the VUmc consists of an easy-to-use algorithm of seven parameters: respiratory rate, saturation rate, heart frequency, systolic blood pressure, temperature, consciousness, urine production (Fig 1). Also, 1 point can be added if a nurse is worried about the patient. If a vital parameter was not documented in the system, this parameter was considered to be normal, therefore 0 points were given. When a patient on a general ward has a score of 3 or higher, the nurse is expected to call the ward doctor on duty or can immediately call the RIT team (Rapid Intervention Team). However, the nurses were not required to perform MEWS on daily basis on set time points and the MEWS was mostly performed on indication which probably led to late detection of the deteriorating patient. It was decided to reintroduce the MEWS protocol in 2015, this study was performed before reimplementation.

### Assessment

For analysis doctor’s charts, nurse’s charts and electronic patient files including all test results were available. Using a standardised abstraction form for each individual ICU-admission, patient characteristics such as the age, mortality, APACHE scores and circumstances under which ICU-admission took place (such as length of stay in the wards and admission speciality) were collected (S1 File) [20–22]. Vital parameters measured and the use of the MEWS-protocol in the 48 hours before the acutely unplanned ICU admission were also systematically registered into this form.

Two medically and PRISMA-trained investigators (BD, PS) reviewed each case extensively and filled out these chart abstraction forms. This analysis was a labour intensive process (approximately 90 minutes average per investigator per chart). Subsequently they composed individual causal trees; thereafter consensus was reached in a structured meeting with a third independent experienced medical and PRISMA reviewer (LG), from which a composite root causal tree was constructed. Finally, all cases and their causal trees were discussed with two senior physicians (PN, JL) and a psychologist with a special interest in PRISMA-analysis (HM), which resulted in the final root causal trees.

### PRISMA-analysis

**Fig 2:**
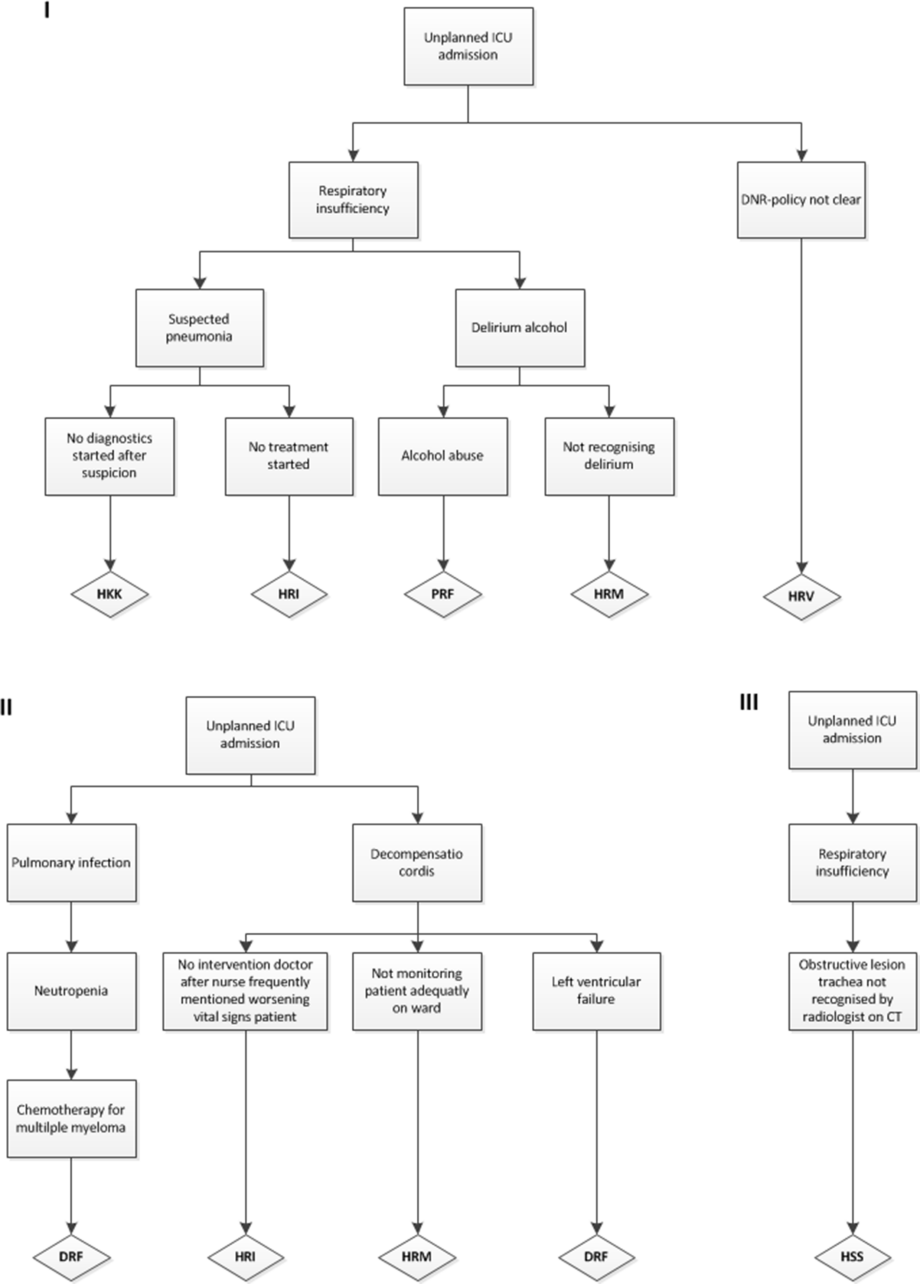
Three examples of root causal trees. HKK: Human-related knowledge behaviour, HRI: Human-related intervention, PRF: Patient-related factor, HRM: Human-related monitoring, HRV: Human-related verification, DRF: Disease-related factor, HSS: Human-related skills-based.

Fig 2 shows three examples of causal trees from this study. In general the PRISMA-method can be used to examine latent factors (technical and organisational), active failures (human) and other factors (patient-related and other). A PRISMA-analysis starts with an incident, in this study the unplanned ICU-admission was seen as the incident. This incident was then placed at the top of the causal tree and was the starting point for the analysis. The next step was to identify the direct causes underlying the unplanned ICU-admission. Direct causes are revealed by asking ‘why’ this incident has occurred. Information from the records was used to identify these direct causes. Below every direct cause the indirect causes were inserted. By constantly asking ‘why’ an event had taken place, relevant indirect causes were systematically exposed. If no more objective information was acknowledged as a cause, the last noted indirect cause was labelled as a root cause and was placed at the bottom of the causal tree. The analysis also stopped when the underlying cause lied outside of the hospital. The root causes were then classified using the Eindhoven Classification Model (ECM), which is the corresponding taxonomy of PRISMA-medical to classify root causes. The ECM is based on the skill-rules-knowledge-based behavioural model of Rasmussen and on the system approach to human error of Reason [23–25]. The ECM was also used as a foundational component in the conceptual framework for the International Classification for Patient Safety [26, 27]. Table 1 shows the classification of the root causes according to the Eindhoven Classification Model [28]. Since previous studies have shown that progression of disease is a valuable addition to this model applied in medical settings, this was added to the root causes for the current study [16].

**Table 1:** Description of categories of the Eindhoven Classification Model: PRISMA-medical Version [17, 18].

Main category	Subcategory	Code	Description	Examples (if available) *
**Technical**	External	T-ex	Technical failures beyond the control of the organisation.	Not available
	Design	TD	Failures to poor design of equipment etc.	Not available
	Construction	TC	Correct design inappropriately constructed or placed.	Not available
	Materials	TM	Material defects not classified under TD or TC.	Not available
**Organisational**	External	O-ex	Failures at an organisational level beyond the control and responsibility of the investigating team.	Not available
	Transfer of knowledge	OK	Failure resulting from inadequate measures to train or supervise new or inexperienced staff.	Not available
	Protocols	OP	Failures relating to the quality or availability of appropriate protocols.	• Not following pain treatment protocol after surgery
	Management priorities	OM	Internal management decisions which reduce focus on patient safety when faced with conflicting priorities.	• No beds available at ICU
	Culture	OC	Failure due to attitude and approach of the treating organisation.	• Ward where vital parameters are not frequently taken since ‘no one does it’
**Human**	External	H-ex	Human failures beyond the control of the organisation/department	• Intoxication of too high dosage medication prescribed outside hospital care (by GP)
	Knowledge-based behavior	HKK	Failure of an individual to apply their knowledge to a new clinical situation	• No adequate diagnostics • No physical examination done
	Qualifications	HRQ	An inappropriately trained individual performing the clinical task	Not available
	Co-ordination	HRC	A lack of task co-ordination within the healthcare team	• No coordination of hypertension treatment
	Verification	HRV	Failure to correctly check and assess the situation before performing interventions	• DNR policy not adequately discussed
	Intervention	HRI	Failure resulting from faulty task planning or performance	• No diagnostics and adequate treatment delirium
	Monitoring	HRM	Failure to monitor the patient’s progress or condition	• No evaluation of vitals after changing treatment • Vitals not monitored and action undertaken after reported deterioration
	Skills-based	HSS	Failure in performance of highly developed skills	• Obstructive lesion trachea not recognised/missed by radiologist on CT
**Patient**	Patient-related	PRF	Failures related to patient characteristics or conditions, which are beyond the control of staff and influence clinical progress	• Monitoring not adequate because patient refused CAD
	Disease-related	DRF	Failures related to the natural progress of disease which are beyond control of patient, its carers and staff	• Tumor progression in vena cava inferior • Biliary pancreatitis
**X**	Unclassifiable	X		• Medication was still being dosed properly • Toxic reaction chemotherapy

*A table with overview of all root causes is provided in S1 Table.

### Statistical analysis

Using IBM SPSS Statistics, Chicago, USA, Version 22.0 descriptive characteristics and frequencies were calculated. Categorical outcome measures are presented as frequencies and percentages. For continuous variables we chose to use median and interquartile ranges since none of them were normally distributed.

## Results

### Patient characteristics

RISMA-analysis was performed on 49 available cases, since one chart was unavailable for review during the study period. Table 2 displays baseline patient characteristics. The median age was 69 years (range 34–90), and both sexes were represented almost equally (47% vs 53%). Nineteen patients died during their hospital admission, resulting in 39% in-hospital mortality. The three scores for ICU-mortality (SAPS II, APACHE II, APACHE IV) had a median of 51, 24, and 95 resulting in a predicted mortality of 50%, 55%, 36% respectively. The median time between hospital admission and ICU admission was 88 hours and 34 minutes (range 1h38 min– 733h). Most patients were transferred to the ICU between midnight and six o’clock in the morning. Sixty-nine percent of the patients (n = 34) had a ‘Do not resuscitate’-policy (DNR), for three patients the DNR-policy was not clear (6%).

**Table 2:** Patient characteristics.

N (%)	49 (100%)
Age—median(range)	69 (34–90)
Male	23 (47%)
Deceased during admission	19 (39%)
Admission specialty: • *Cardiology* • *Gastro-enterology* • *Hematology* • *Internal medicine* • *ENT-diseases* • *Pulmonary medicine* • *Neurology* • *Nephrology* • *Oncology* • *Traumatology* • *Vascular surgery*	• 1 (2%) • 5 (10%) • 7 (14%) • 12 (24%) • 1 (2%) • 9 (18%) • 3 (6%) • 3 (6%) • 3 (6%) • 4 (8%) • 1 (2%)
Polypharmacy*	36 (73)
Length of stay before unplanned transfer ICU in hours–median (range)	88 h 34 m (1h38m-733h)
Time unplanned ICU admission • *24*.*00–06*.*00* • *06*.*00–12*.*00* • *12*.*00–18*.*00* • *18*.*00–24*.*00*	• 17 (35%) • 12 (24%) • 11 (22%) • 9 (18%)
DNR-policy before ICU admission • *No restriction* • *Do not rescuscitate*, *do ventilate* • *Do not rescuscitate*, *do not ventilate* • *No ICU admission* • *Not clear*	• 34 (69%) • 9 (18%) • 3 (6%) • 0 (0%) • 3 (6%)
SAPS II–median(range)[21]	51 (18–110)
APACHE II–median (range) [20]	24 (6–45)
APACHE IV–median (range) [22]	95 (36–186)

*The concomitant use of five or more drugs.

### Root causes

**Fig 3:**
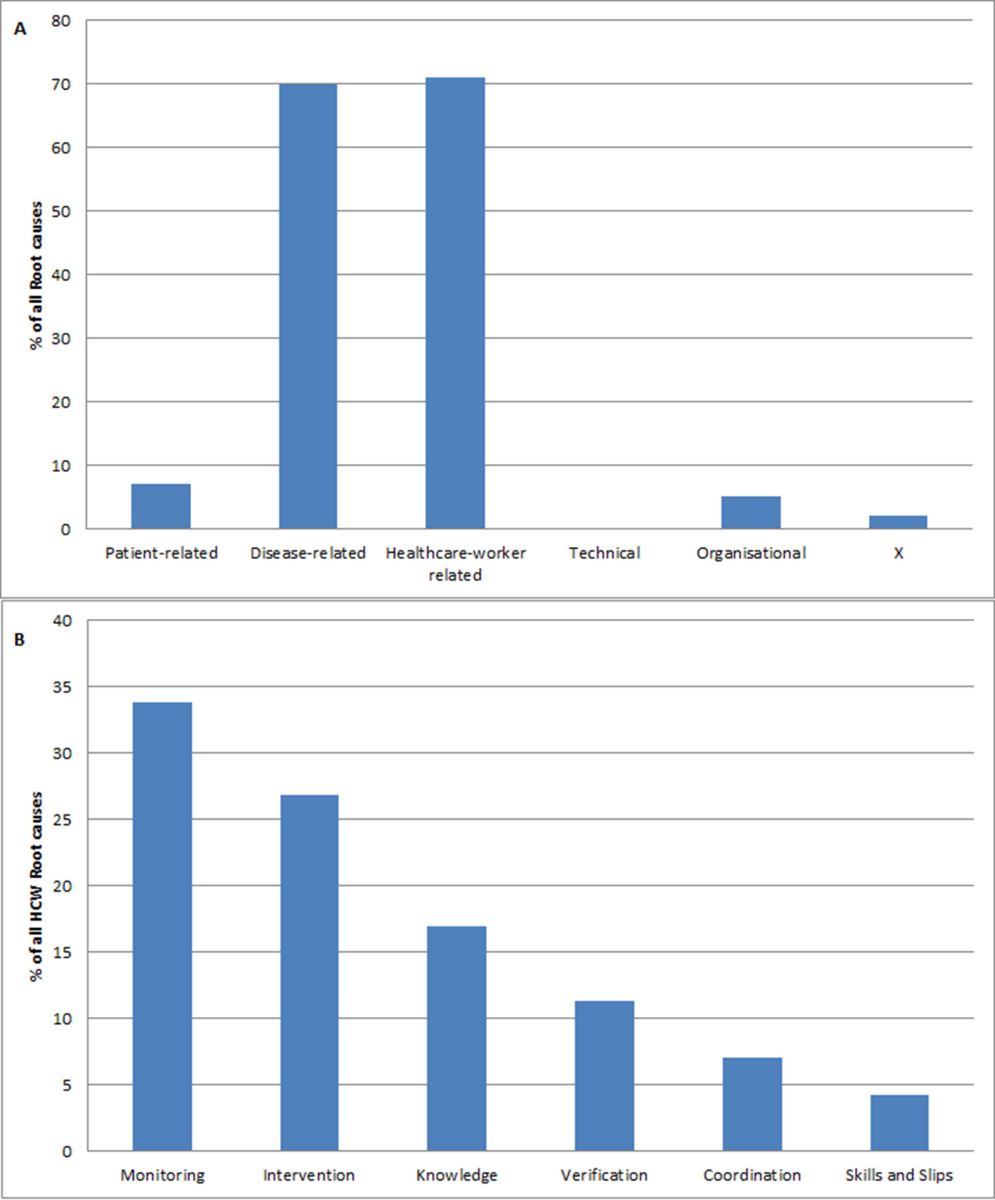
Distribution root causes. 3a Main categories root causes. 3b Healthcare worker (HCW) root causes.

In total, from 49 unplanned ICU causal trees, 155 root causes were identified after PRISMA-analysis. See Fig 2 for three of the composed causal trees. Twelve unplanned ICU admissions had one root cause (22%), 7 (14%) had two root causes, 11 (22%) three root causes, 12 four root causes (25%) and 2 cases had 5 root causes (4%). Twelve percent of the cases had six or more root causes (n = 6). Almost half of the root causes identified were disease-related (n = 70, 45%). Forty-four cases had at least one disease-related root cause. These disease-related root causes comprise failures which are related to the natural progression of a disease which are beyond control of patient, its carers and staff, for example neutropenic sepsis in a patient with chemotherapy for an hematologic disease. Table 1 illustrates the subcategories used in PRISMA-analysis with examples from our study. The distribution of all the root causes is illustrated in Fig 3A.

Almost half of the root causes classified were human-related (n = 71, 46%), followed by patient-related causes (n = 7, 5%), and organisational root causes (n = 5, 3%). Human related causes are causes which are healthcare worker related. Most of the healthcare worker related causes (34%) were monitoring failures (HRM), these are. failures in monitoring the patient’s progress or condition, e.g. not monitoring vital parameters in a patient who is clinically deteriorating. Fig 3B shows the subcategorisation of healthcare worker root causes. Human intervention causes were accountable for almost one-third (27%) of the causes. They represent failures resulting from faulty task planning or performance, for example the case where no intervention was started after the nurse repeatedly mentioned patient’s vital signs worsening. The remaining human-related root causes were verification (HRV: 11,3%), coordination (HRC: 7%), and skills-based (HSS: 4,2%) failures. Finally, the organisation root causes were those caused by management issues, such as lack of beds on the ICU, or not handling certain treatment according to the institutional protocol. The two unclassifiable root causes are shown in Table 1. No technical related root causes (T) were identified in this PRISMA-analysis.

### Use of Track and Trigger System/Early warning score

Table 3 shows that in 86% (n = 42 patients) of the cases orders were given by the doctors for vital parameter monitoring. This was done as agreed in only half of the cases (n = 20, 49%). In the 48 hours before ICU admission, a total of 477 vital parameter sets were measured in the 49 patients (i.e. 1 vital set could include: a blood pressure, pulse, breathing frequency and temperature), with a median of 6 sets measured per patient. Of these 477 sets, an explicit MEWS was calculated and documented correctly, according to protocol, in only 6 of these measurements in 4 patients (1%). After recalculation by the researcher based upon the available vital parameters, 207 (43%) of the vital sets gave a critical MEWS score of 3 or higher. 46 patients had at least one critical MEWS of 3 or higher during the 48 hours before ICU admission. According to MEWS protocol, this implied that a doctor had to be called. In 125 of the measured sets, in 42 patients, this was done (although the MEWS score was often not calculated and documented), and after this the doctor started an action upon this in 117 of these 125 phone calls. In total, 67 of all measurements, in 92% of the patients (n = 45), led to a call to the RIT at least once.

**Table 3:** Use of Track and Trigger system.

Vital parameters documentation	Frequency (%), N = 49 (100%)	
Orders were given for vital monitoring*	42 (86%)	
Vital monitoring performed as agreed	20 (41%)	
Registration of ICU admission in nurse’ charts	32 (65%)	
Registration of ICU admission in doctors’ charts	38 (78%)	
	
**MEWS Documentation**		
Total vital set measurements done in 48 hours before ICU admission in 49 patients	N = 477 (100%)	
Number of vital set measurement done per patient in 48 hours before ICU admission–median(range)	6 (1–22)	
Doctor called after vital parameters measured	174 (36%)	42 patients
Doctor started an action after being called	164 (34%)	42 patients
Evaluation after 60 minutes of started action	96 (20%)	41 patients
RIT-call	69 (14%)	45 patients
MEWS calculated and documented correctly in charts	6 (1%)	4 patients
Critical MEWS (after recalculation by researcher using vital set measurements ≥3)	207 (43%)	46 patients
Doctor called at a critical-MEWS according to the recalculated score by the researcher	125 (26%)	42 patients

*Vital monitoring: arrangements about frequency and type of vital set measurements to be done by nurses on the wards.

## Discussion

The current study is the first to systematically investigate the root causes of unplanned ICU admissions. The most important finding from this study was that almost half of the root causes contributing to unplanned ICU admission were human (healthcare worker) related. These causes predominantly included human monitoring and intervention failures, indicating flaws in monitoring the patients progress or condition and faulty task planning or performance. This illustrates a potential for improvement. The other half of the root causes were disease-related, comprising the root causes related to the natural progression of the disease, which was to be expected in this overall severely ill patient population, as reflected in their high mortality rates. The secondary aim was to further investigate monitoring by analysing the use of a TTS on a general ward in the early recognition of deteriorating patients. Results showed that in the 48 hours before deterioration only in 1% of the measured vital sets an explicit correct ‘MEWS’ was reported, although in 43% of the measurements patients had a critical score. This is an important clinical finding, since it seems essential that recognition of critical scores by hospital staff is present in order to improve monitoring [29].

Monitoring failures emerging from the PRISMA-analysis were diverse. One example is a nurse who documented in the chart that a patient repeatedly complains about shortness of breath, did not undertake an action to measure vital parameters or request a physician for assessment. Another example was unclear handovers resulted in confusion about how intensively the patient needed monitoring, resulting in inadequate and insufficient monitoring measurements. Interventional problems are exemplified by not performing adequate diagnostics and treatment in a severely immunosuppressed patient with a suspected pneumonia. In our study, an unclear DNR-policy was also found to be responsible for an unnecessary unplanned ICU admission in a few cases, since these patients did not want to be admitted to the ICU but this was not known or discussed on the ward. These are human-related verification failures (HRV).

The above results seem to be consistent with earlier research indicating that unplanned ICU admissions are not only caused by the underlying disease, but are also potentially caused by suboptimal care on the ward, and by inadequate assessment and monitoring of a patients status [30, 31]. Other studies have shown that certain patient characteristics such as older age, being male and having a higher co-morbidity increase the chance of unplanned ICU admissions [32]. In addition to above mentioned non-modifiable factors, our study results provides implications for practice to potentially reduce unplanned ICU admission by improving patient monitoring.

One method for improving the recognition of these patients, is the implementation of TTSs. Although the conclusions on effectiveness of TTSs in reducing clinical endpoints are still not uniform, when properly followed they are effective in identifying deteriorating patients [33, 34]. The effectiveness depends on appropriate implementation, compliance and an effective clinical response [35]. The current study has shown that the MEWS score in the studied population was registered according to protocol in very few patients. For the other patients, a score was calculated by the researchers based on the available vital parameters. After recalculation 43% (207 out of 477) of the measurements were critical, and in these cases in 60% (125 out of 207 measurements) a doctor was called in although an explicit MEWS was not documented in the charts. If the doctor was notified, in most cases they started an action, which shows the relevance of these critical measurements. These results show that the implementation of MEWS at the time of the study was insufficient. When we implemented the protocol a few years back the nurses and doctors were requested to perform MEWS when they found patients to be sick and in need of critical care. The protocol did not require the MEWS to be taken daily on set times to identify deterioration early. The added value of the daily measurements has been demonstrated [35]. We therefore implemented a new protocol in which it was compulsory to measure the MEWS in all patients at least once a day in the morning. If the MEWS is >3 the doctor has to be called within 30 minutes. The clinical staff was (re)trained aiming to chance their mind-set about the importance of MEWS. A few weeks after this implementation the protocol adherence improved to 89%, underlining the importance of robust implementation [36].

This in-depth study has provided analysis of the root causes of unplanned ICU admissions to the full extent. To our knowledge, this is the first study performed that explores the underlying causes of unplanned ICU admissions in such a way that it provides clear insight in the areas for potential improvement in the healthcare system. Hence, it equips us with valuable information about areas in need of attention. From this, tools for implementing process improvement in hospitals can be initialised. A limitation of the study was its retrospective nature, which means that researchers had to rely on written information in the patient records, instead of actively interviewing the persons involved. Therefore valuable information might have been missed and this could potentially have led to an underestimation of the factors leading to delayed recognition. For future studies it is therefore recommended to combine this research method with other methods, such as collecting qualitative data using interviews. Also, by assessing these cases only on the basis of information available on paper, analysis might not elucidate all the organisational factors which are not typically written down and are latent.

## Conclusion

This study is the first one that has systematically provided insight in the root causes of unplanned ICU admissions. Half of the root causes identified were healthcare worker related failures, mainly resulting from monitoring and interventional failures. The monitoring of patients might be improved by a properly functioning Track and Trigger System. Results in this study however, have shown that this warrants improvement.

## Supporting Information

S1 Dataset ICU admissions. (SAV)

S1 File Data collection sheet unplanned ICU admissions. (DOC)

S1 Table Overview root causes. (DOCX)
