# Supplementary material for: Delayed Recognition of Deterioration of Patients in General Wards Is Mostly Caused by Human Related Monitoring Failures: A Root Cause Analysis of Unplanned ICU Admissions
Source: PLoS One. 2016 Aug 18;11(8):e0161393. doi: 10.1371/journal.pone.0161393 (PMC4990328; doi:10.1371/journal.pone.0161393)
Supplement: S1 File — (DOC) [file pone.0161393.s002.doc]

**S1 File: Data collection sheet unplanned ICU admissions**

**(English version)**


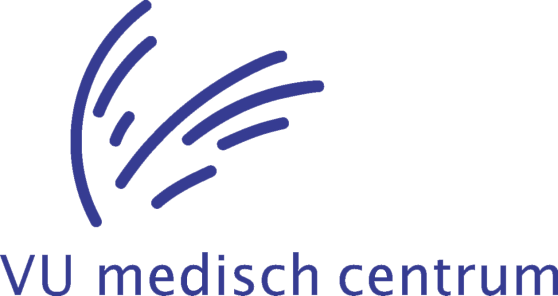


Vrije Universiteit medisch centrum

Postbus 7057

1007 MB Amsterdam

telefoon 020 44 44 444

fax 020 44 44 645

# **Unplanned ICU Admissions**

# Data collection sheet chart review

# 2016

Copyright © 2016. All rights reserved. No part of this questionnaire may be reproduced, stored in a automatized database or made public in any form or by any means (electronic, mechanical, photocopying, recording or otherwise), without the prior written consent of the principal investigator.

**A DATA REVIEWER**

1 Date of reviewing:

2 Start time rating (hours and minutes):

3 Name assessor:

4 Stop time rating (hours and minutes)

**B UNPLANNED ICU ADMISSION AND MEWS**

**Patient and admission characteristics:**

1. Patient deceased:

- Yes
- During this admission
- Not during this admission
- No

1. Date admission hospital:
2. Reason for admission hospital:
3. Date admission ICU:
4. Was a DNR-policy clear before ICU admission (if yes, what is this)?
5. Did the patient use 5 or more medicine at home before admission (polypharmacy)?

- Yes
- No

1. Was medication use at the ward changed before ICU admission?

- Yes
- No

1. Was an admission passport present?

- Yes
- No

1. Were arrangements made about vital monitoring between doctor and nurses?

- Yes
- No

1. Were vital parameters measured as agreed?

- Yes
- No

1. Did the nurse mention the ICU-admission in the chart?

- Yes
- No

1. Did the physician on the ward mention the ICU admission in the chart?

- Yes
- No

**MEWS (< 48 uur before unplanned ICU admission)**

Note all vital parameters taken in the 48 hours before the unplanned ICU admission.

**MEWS (< 48 uur before unplanned ICU admission)**

| **Date** | **Time** | **Type of day** | **HF** | **Sys RR** | **AF** | **Temp** | **AVPU** | **Worried** | **Urine** | **Sat +/- O_2_** | **MEWS** | **Mews noted** | **RIT call** | **Doctor called** | **Treatment < 30min** | **Evaluation treatment < 60min** |
| --- | --- | --- | --- | --- | --- | --- | --- | --- | --- | --- | --- | --- | --- | --- | --- | --- |
|  | __ : __ | O/A/E/G |  |  |  |  |  | Yes / No | _ |  | __ | __ |  | Yes / No | Yes / No | Yes / No | Yes / No | Yes / No |
|  | __ : __ | O/A/E/G |  |  |  |  |  | Yes / No | _ |  | __ | __ |  | Yes / No | Yes / No | Yes / No | Yes / No | Yes / No |
|  | __ : __ | O/A/E/G |  |  |  |  |  | Yes / No | _ |  | __ | __ |  | Yes / No | Yes / No | Yes / No | Yes / No | Yes / No |
|  | __ : __ | O/A/E/G |  |  |  |  |  | Yes / No | _ |  | __ | __ |  | Yes / No | Yes / No | Yes / No | Yes / No | Yes / No |
|  | __ : __ | O/A/E/G |  |  |  |  |  | Yes / No | _ |  | __ | __ |  | Yes / No | Yes / No | Yes / No | Yes / No | Yes / No |
|  | __ : __ | O/A/E/G |  |  |  |  |  | Yes / No | _ |  | __ | __ |  | Yes / No | Yes / No | Yes / No | Yes / No | Yes / No |
|  | __ : __ | O/A/E/G |  |  |  |  |  | Yes / No | _ |  | __ | __ |  | Yes / No | Yes / No | Yes / No | Yes / No | Yes / No |
|  | __ : __ | O/A/E/G |  |  |  |  |  | Yes / No | _ |  | __ | __ |  | Yes / No | Yes / No | Yes / No | Yes / No | Yes / No |
|  | __ : __ | O/A/E/G |  |  |  |  |  | Yes / No | _ |  | __ | __ |  | Yes / No | Yes / No | Yes / No | Yes / No | Yes / No |
|  | __ : __ | O/A/E/G |  |  |  |  |  | Yes / No | _ |  | __ | __ |  | Yes / No | Yes / No | Yes / No | Yes / No | Yes / No |
|  | __ : __ | O/A/E/G |  |  |  |  |  | Yes / No | _ |  | __ | __ |  | Yes / No | Yes / No | Yes / No | Yes / No | Yes / No |
|  | __ : __ | O/A/E/G |  |  |  |  |  | Yes / No | _ |  | __ | __ |  | Yes / No | Yes / No | Yes / No | Yes / No | Yes / No |
|  | __ : __ | O/A/E/G |  |  |  |  |  | Yes / No | _ |  | __ | __ |  | Yes / No | Yes / No | Yes / No | Yes / No | Yes / No |
|  | __ : __ | O/A/E/G |  |  |  |  |  | Yes / No | _ |  | __ | __ |  | Yes / No | Yes / No | Yes / No | Yes / No | Yes / No |
|  | __ : __ | O/A/E/G |  |  |  |  |  | Yes / No | _ |  | __ | __ |  | Yes / No | Yes / No | Yes / No | Yes / No | Yes / No |
| **Date** | **Time** | **Time of day** | **HF** | **Sys RR** | **AF** | **Temp** | **AVPU** | **Worried** | **Urine** | **Sat +/- O_2_** | **MEWS** | **Mews noted** | **RIT call** | **Doctor called** | **Treatment < 30min** | **Evaluation treatment < 60min** |
|  | __ : __ | O/A/E/G |  |  |  |  |  | Yes / No | _ |  | __ | __ |  | Yes / No | Yes / No | Yes / No | Yes / No | Yes / No |
|  | __ : __ | O/A/E/G |  |  |  |  |  | Yes / No | _ |  | __ | __ |  | Yes / No | Yes / No | Yes / No | Yes / No | Yes / No |
|  | __ : __ | O/A/E/G |  |  |  |  |  | Yes / No | _ |  | __ | __ |  | Yes / No | Yes / No | Yes / No | Yes / No | Yes / No |
|  | __ : __ | O/A/E/G |  |  |  |  |  | Yes / No | _ |  | __ | __ |  | Yes / No | Yes / No | Yes / No | Yes / No | Yes / No |
|  | __ : __ | O/A/E/G |  |  |  |  |  | Yes / No | _ |  | __ | __ |  | Yes / No | Yes / No | Yes / No | Yes / No | Yes / No |
|  | __ : __ | O/A/E/G |  |  |  |  |  | Yes / No | _ |  | __ | __ |  | Yes / No | Yes / No | Yes / No | Yes / No | Yes / No |
|  | __ : __ | O/A/E/G |  |  |  |  |  | Yes / No | _ |  | __ | __ |  | Yes / No | Yes / No | Yes / No | Yes / No | Yes / No |
|  | __ : __ | O/A/E/G |  |  |  |  |  | Yes / No | _ |  | __ | __ |  | Yes / No | Yes / No | Yes / No | Yes / No | Yes / No |
|  | __ : __ | O/A/E/G |  |  |  |  |  | Yes / No | _ |  | __ | __ |  | Yes / No | Yes / No | Yes / No | Yes / No | Yes / No |
|  | __ : __ | O/A/E/G |  |  |  |  |  | Yes / No | _ |  | __ | __ |  | Yes / No | Yes / No | Yes / No | Yes / No | Yes / No |
|  | __ : __ | O/A/E/G |  |  |  |  |  | Yes / No | _ |  | __ | __ |  | Yes / No | Yes / No | Yes / No | Yes / No | Yes / No |
|  | __ : __ | O/A/E/G |  |  |  |  |  | Yes / No | _ |  | __ | __ |  | Yes / No | Yes / No | Yes / No | Yes / No | Yes / No |
|  | __ : __ | O/A/E/G |  |  |  |  |  | Yes / No | _ |  | __ | __ |  | Yes / No | Yes / No | Yes / No | Yes / No | Yes / No |
|  | __ : __ | O/A/E/G |  |  |  |  |  | Yes / No | _ |  | __ | __ |  | Yes / No | Yes / No | Yes / No | Yes / No | Yes / No |

Instructions:

1. When no vital parameter is measured, leave space blank.

2. When vital parameter is not clear or not readable, fill in 999

3. Saturation: always report if patient was on additional oxygen

4. Type of day: **O: Admission day** **A: Ward day** **E: Event day G: No measurements on this day**

**C identifY CAUSES of unplanned ICU admissions**

**Description incident:**

1 Describe the reasons for the unplanned ICU admission.

2 Describe the direct causes that resulted in the unplanned ICU admission.

3 Please note below the underlying causes for the direct causes by repeating the "why"question. Stop if there are no more objective facts , but just opinions or guesses.

4 Derived from 3., what are the root causes for this readmission?

5 Is the unplanned ICU admission related to the admission on the ward?

6 Is there any reason to think that the unplanned ICU admission potentially could have been prevented? (if yes, please explain)

7 Draw the causal tree on the next page completely using the answers to previous questions. As a guide use to the example of a causal tree in Appendix I [van der Schaaf]. Use "and" ports if the reasons certainly contributed to the occurrence of a re-admission. Use an "or" port when the causes potentially contributed to the reason for readmission.

**Causal tree**

| UNPLANNED ICU ADMISSION |
| --- |

Medical version of the Eindhoven Classification Model
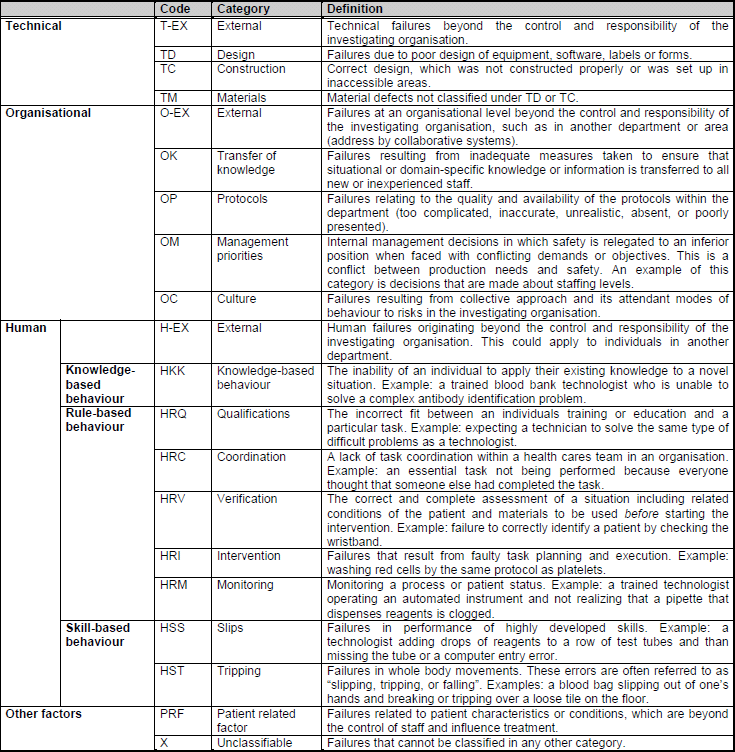
 (van Vuuren et al., 1997)
