# Supplementary material for: Delayed Recognition of Deterioration of Patients in General Wards Is Mostly Caused by Human Related Monitoring Failures: A Root Cause Analysis of Unplanned ICU Admissions
Source: PLoS One. 2016 Aug 18;11(8):e0161393. doi: 10.1371/journal.pone.0161393 (PMC4990328; doi:10.1371/journal.pone.0161393)
Supplement: S1 Table — (DOCX) [file pone.0161393.s003.docx]

| **DRF** | **Total 71** |
| --- | --- |
|  | Active fluid resuscitation in patient with heart failure |
|  | Sepsis osteosynthesis knee |
|  | Hypertensive crisis |
|  | Pneumonia |
|  | COPD |
|  | Tumor progression in vena cava inferior |
|  | Myasthenia gravis crisis |
|  | Aspiration pneumonia |
|  | Medicament renal failure |
|  | Pre-existent vascular and cardiac failure |
|  | FSGS |
|  | Neutropenia after chemotherapy |
|  | Perforation colon after chemotherapy |
|  | Pulmonary embolism |
|  | SLE |
|  | Pleural infusion |
|  | Decompensatio Cordis |
|  | Pleural effusion |
|  | Seam leakage after operation |
|  | Pneumonia and super infection |
|  | Diverticular abces |
|  | Prednison / MTX |
|  | Demand ischemia |
|  | Urosepsis |
|  | Myositis |
|  | Ischemic CVA during dialysis |
|  | Atriumfibrillation de novo |
|  | Left Ventriculair Dysfunction |
|  | Immunocompromised |
|  | Anemia |
|  | SIADH |
|  | Sintrom use |
|  | Encephalitis |
|  | Post-OK complication, adequately treated |
|  | Metastatic melanoma |
|  | Pneumosepsis |
|  | Component COPD |
|  | Lesion right lung |
|  | AF de novo |
|  | Interstitial pulmonary disorder |
|  | Hepatic encephalopathy livercirrhosis |
|  | Astma cardiale/renale because of prehydration CT – scan, protocol well followed |
|  | Pneumonia |
|  | Pre-existent memory loss |
|  | Sepsis in immunocompromised patient eci |
|  | GVHD with Non-Hodgekin Lymfoma |
|  | Bilateral pneumoniae |
|  | Heart failure |
|  | Pneumonia with immunocompromised patient |
|  | Prednison for brain tumor |
|  | Decompensatio cordis |
|  | Urosepsis |
|  | Neutropenia due to chemo for multiple myeloma |
|  | Left ventriculair failure |
|  | Pulmonary embolism |
|  | Medication for hematologic transplantation |
|  | Sepsis eci |
|  | Dehydration with diarrhea |
|  | Distributive shock (sepsis?) |
|  | Billiary pancreatitis |
|  | Klebsiella Pneumonia |
|  | HSV1-virus |
|  | Pneumonia immunosuppressed patient due to chemotherapy |
|  | Legionella pneumonia |
|  | Pre and post hydration elective CT, patient had heart failure but this was not known yet |
|  | Cholangitis |
|  | E. coli pneumonia |
|  | Pulmonary hypertension with heart failure |
|  | Pneumonia |
|  | Hematologic disease |

| **PRF** | **Total 7** |
| --- | --- |
|  | Monitoring not adequate because patient refused CAD |
|  | Wish patient not to flush drain because of pain |
|  | No adequate therapy commitment patient hypertension |
|  | Alcoholabuse |
|  | Delirium alcohol |
|  | Unclear DNR policy, patient not clear what she wanted |
|  | Relative intoxication carbamazepine, with less intake at home |

| **HRM** | **Total 24** |
| --- | --- |
|  | 16 hours before ICU admission MEWS score kept getting higher without consultation doctor |
|  | No evaluation of vitals after changing treatment |
|  | Vital signs nog adequately monitored |
|  | Vitals not monitored adequately after order doctor requested this |
|  | No vitals monitored |
|  | No adequate reaction to vital parameters worsening |
|  | Not monitored decompensatio cordis |
|  | No vitals reported |
|  | No clear handovers around patient |
|  | No coordination of hypertension treatment |
|  | Patient not adequately monitored on ward |
|  | Ward and doctor policy on monitoring vital parameters on the ward |
|  | No diagnostics and adequate treatment delirium |
|  | Fluid resuscitation not adequately monitored patient with known heart failure |
|  | Vitals not adequately monitored after fluid resuscitation |
|  | Vitals not monitored adequately |
|  | No physical examination done |
|  | Vitals not adequately monitored, patient continuously says she is short of breath |
|  | No monitoring after medication change (Lasix) |
|  | Vitals not adequately monitored after fluid resuscitation |
|  | Not monitoring patient adequately |
|  | Vital signs not monitored or reported |
|  | Vitals not monitored and action undertaken after reported deterioration |
|  | Nurse did not monitor patient adequately as ordered by doctor |

| **HRI** | **Total 19** |
| --- | --- |
|  | Antibiotics not given at ward, no prescription, and no verification check by nurse |
|  | No intervention after reported weight gain |
|  | Vitals not monitored adequately after order doctor requested this |
|  | Delayed diagnosis and antibiotic treatment |
|  | No adequate reaction to vital parameters worsening |
|  | No adequate reaction to lower vital signs |
|  | Pain abdomen not adequately recognised and treatment |
|  | No adequate treatment with suspicion |
|  | No diagnostics and adequate treatment delirium |
|  | No adequate pain treatment after OK |
|  | Antihypertensiva not stopped during admission |
|  | Intoxication benzo’s with livercirrhosis |
|  | Vitals not adequately taken by nurses after orders doctor |
|  | No intervention doctor after signs patient frequently mentioned by nurse |
|  | Vitals not monitored and action undertaken after reported deterioration |
|  | No adequate intervention when vital signs worsen |
|  | No action performed after deterioration noticed |
|  | Patient should have gone to ICU immediately |
|  | No intervention doctors, after vital signs monitored and worsening |

| **HRV** | **Total 8** |
| --- | --- |
|  | Antibiotics not given at ward, no prescription, and no verification check by nurse |
|  | DNR policy not adequately discussed |
|  | No physical examination done |
|  | No diagnostics and adequate treatment delirium |
|  | Not clear DNR policy |
|  | Vitals not adequately taken by nurses after orders doctor |
|  | DNR policy not adequately discussed |
|  | Prednison started too late |

| **HRC** | **Total 5** |
| --- | --- |
|  | Nurse called doctor because she thought patient was decompensated and wanted action didn’t happen, no report of this noted |
|  | Pain abdomen not adequately recognised and treatment |
|  | Vital signs not monitored as ordered |
|  | No coordination of hypertension treatment |
|  | No clear orders about monitoring patient |

| **HKK** | **Total 12** |
| --- | --- |
|  | No adequate diagnostics fever |
|  | Antibiotics after operation not given even though patient had sepsis pre OK |
|  | Intensivist didn’t think patient was in need of ICU admission |
|  | Closing tracheostoma against medical advice specialist |
|  | No adequate diagnostics, no physical exam etc. |
|  | Antihypertensiva not stopped during admission |
|  | Fluid resuscitation not adequately monitored patient with known heart failure |
|  | Intoxication benzo’s with livercirrhosis |
|  | Too much fluids with medical history of heart failure |
|  | Nurse doesn’t measure breath frequency and other controls when inquired |
|  | No diagnostics performed etiology shock/ sepsis |
|  | Patient should have gone to ICU immediately |

| **HSS** | **Total 3** |
| --- | --- |
|  | After suction with prolonged bleeding time with livercirrhosis |
|  | Missed diagnosis by radiologist |
|  | Obstructive lesion trachea not recognised by radiologist on CT |

| **HEX** | **Total 1** |
| --- | --- |
|  | Medication Intoxication outside hospital care (GP and psychiatrist) |

| **OM** | **Total 3** |
| --- | --- |
|  | Delay since there was no place at MC |
|  | No beds available at ICU |
|  | No space medium care, nurses wanted patient to be transferred. ICU doctor said no space MC |

| **OC** | **Total 1** |
| --- | --- |
|  | Ward and doctor policy on monitoring vital parameters on the ward |

| **OP** | **Total 1** |
| --- | --- |
|  | No adequate pain treatment after OK |

| **X** | **Total 2** |
| --- | --- |
|  | Medication was still being dosed properly |
|  | Toxic reaction chemotherapy |
